# Supplementary material for: Causal Relationship between Aging and Anorexia Nervosa: A White-Matter-Microstructure-Mediated Mendelian Randomization Analysis
Source: Biomedicines. 2024 Aug 16;12(8):1874. doi: 10.3390/biomedicines12081874 (PMC11351342; doi:10.3390/biomedicines12081874)
Supplement: Supplementary file 1 [file biomedicines-12-01874-s001.zip › Supplementary Figure/Figure S8/Leaveoneout plot of MR analysis for TL to ubm-b-1480.pdf]

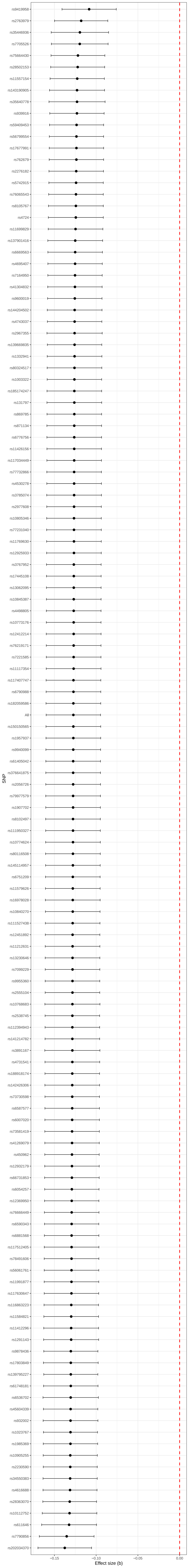

rs9419958
rs2763979
rs35446936
rs7705526
rs75664430
rs28502153
rs11557154
rs143190905
rs35640778
rs939916
rs59409453
rs56799554
rs17677991
rs762679
rs2276182
rs5742915
rs76065543
rs8105767
rs4724
rs11699829
rs137901416
rs6669563
rs4695407
rs7164950
rs41304832
rs9600019
rs144204502
rs4743037
rs2967355
rs139669835
rs1332941
rs80324517
rs1003322
rs185174247
rs131797
rs869785
rs871134
rs6776756
rs11426156
rs117034449
rs77732866
rs4530278
rs3785074
rs2977608
rs10905346
rs77231040
rs11769630
rs12925933
rs3767952
rs17445108
rs13062095
rs10845387
rs4498805
rs10773176
rs12412214
rs76219171
rs7221585
rs11117354
rs117407747
rs6790988
rs182059586
A8
rs150150565
rs1957937
rs9940099
rs61405042
rs376641875
rs2056726
rs79977579
rs1907702
rs8102497
rs111950327
rs10774624
rs80116508
rs145114957
rs6751209
rs11579626
rs16978028
rs10840270
rs111527438
rs12451892
rs11212631
rs13230646
rs7099229
rs9955360
rs2555104
rs10768683
rs2538745
rs112394943
rs141214782
rs3891167
rs4731541
rs188918174
rs142426306
rs73730598
rs6587577
rs6007020
rs73581419
rs41269079
rs450962
rs12932179
rs66731853
rs6054257
rs12369950
rs76666449
rs6590343
rs6881568
rs117512405
rs78491606
rs56061761
rs11991877
rs117630647
rs116863223
rs11584821
rs11412296
rs1291143
rs9878436
rs17803849
rs139795227
rs61748181
rs6536702
rs45604339
rs932002
rs1023767
rs1985369
rs10905255
rs2230590
rs34550383
rs4616688
rs28363070
rs10112752
rs611646
rs7790856
rs202034370
SNP
-0.15
-0.10
-0.05
0.00
Effect size (b)
